# Voxel-based Specific Regional Analysis System for Alzheimer’s Disease and Arterial Spin Labeling in Brain Magnetic Resonance Imaging: A Comparative Study

**DOI:** 10.2174/0115734056337828250526070304

**Published:** 2025-07-10

**Authors:** Yukinori Okada, Norikazu Ohno, Yoshisuke Kitahara, Hirofumi Niioka, Koji Tanaka, Hiromitsu Ueda, Katsunori Tsujii, Masayuki Sato

**Affiliations:** 1 Department of Radiology, Iga City General Hospital Iga, 831 Shijukucho, Iga, Mie 518-0823, Japan; 2 Department of Radiology, Tokyo Medical University, Tokyo, Japan; 3 Department of Neurology, Ohno Clinic, Iga, Japan; 4 Department of Neurology, Iga City General Hospital Iga, 831 Shijukucho, Iga, Mie 518-0823, Japan; 5 Department of Neurosurgery, Iga City General Hospital Iga, 831 Shijukucho, Iga, Mie 518-0823, Japan; 6 Department of Surgery, Iga City General Hospital Iga, 831 Shijukucho, Iga, Mie 518-0823, Japan; 7 Department of Radiological Technology, Iga City General Hospital Iga, 831 Shijukucho, Iga, Mie 518-0823, Japan; 8 Department of Neurology, Hospital, National Center for Geriatrics and Gerontology, Aichi, Japan

**Keywords:** Alzheimer’s disease, Lewy body disease, Brain MRI, Arterial spin labeling, Voxel-based specific regional analysis system for Alzheimer’s disease, Posterior cingulate gyrus

## Abstract

**Introduction:**

Magnetic resonance imaging can differentiate Alzheimer-type dementia from dementia with Lewy bodies using voxel-based specific regional analysis systems for Alzheimer’s disease and arterial spin labeling, which reveal reduced blood flow from the posterior cingulate gyrus to the precuneus in Alzheimer-type dementia. However, the relationship between voxel-based specific regional analysis system scores and arterial spin labeling remains unclear. To investigate the relationship between brain atrophy scores and arterial spin labeling values in the posterior cingulate precuneus.

**Methods:**

Participants with suspected dementia who underwent brain magnetic resonance imaging using a voxel-based regional analysis system were included. They were classified as follows: Group 1 (suspected Alzheimer-type dementia) had atrophy ≥2 in the volume of interest; Group 2 (suspected dementia with Lewy body) had atrophy <2 in the volume of interest and ≥0.2 in the gray and white matter of the dorsal brainstem; and Group 3 included those not meeting these criteria. Correlation values among atrophy within the volume of interest, percentage of atrophic areas, atrophy ratio, percentage of total brain atrophy, age, and maximum arterial spin labeling value at the posterior cingulate precuneus were evaluated.

**Results:**

Groups 1, 2, and 3 comprised 179, 143, and 197 patients, respectively. Arterial spin labeling values at the posterior cingulate precuneus were 77.0±24.4–77.3±25.2, 78.3±81.3–80.2±23.6, and 80.2±22.3–80.4±22.8 mL/min/100 g, respectively. Group 1 had a correlation coefficient between total brain atrophy and arterial spin labeling of –0.189 to–0.214 (P<0.01). Group 2 had a correlation coefficient between total brain atrophy and arterial spin labeling of –0.215 to –0.223 (P<0.01). Group 3 showed no significant correlations. No statistically significant difference was observed in ASL 1 and 2 values between the Alzheimer-type dementia and other groups (ASL 1: 74.5 mL/min/100 g *vs*. 78.8 mL/min/100 g, P=0.08; ASL 2: 74.8 mL/min/100 g *vs*. 79.2 mL/min/100 g, P=0.101). No statistically significant difference was observed in ASL 1 and 2 values between the Alzheimer-type dementia and DLB groups (ASL 1: 74.5 mL/min/100 g *vs*. 69.3. mL/min/100 g, P=0.093; ASL 2: 74.8 mL/min/100 g *vs*. 78.9 mL/min/100 g, P=0.258).

**Discussion:**

Reduced blood flow in the posterior cingulate gyrus and precuneus shows only a weak correlation with brain atrophy in both Alzheimer-type dementia and dementia with Lewy bodies. Therefore, it is not a reliable marker for differentiating Alzheimer-type dementia from dementia with Lewy bodies and other groups.

**Conclusion:**

It is necessary to avoid using cerebral blood flow assessment alone when diagnosing dementia.

## 1 INTRODUCTION

In dementia, cognitive function deteriorates to the extent that it interferes with activities of daily living [1]. Dementia is diagnosed based on physical examination findings, with particular emphasis on psychological tests such as the Mini-Mental State Examination (MMSE), Hasegawa’s Dementia Scale-Revised, and Clinical Dementia Rating. Additionally, imaging modalities, including computed tomography (CT), magnetic resonance imaging (MRI), and single-photon emission CT (SPECT), are used to evaluate dementia. However, the National Health Insurance system in Japan does not cover positron emission CT (PET) for dementia treatment, and the information obtained from head CT is limited to morphological content. Consequently, imaging diagnosis of dementia in Japan is primarily performed using brain MRI and brain perfusion SPECT.

On brain perfusion SPECT, blood flow from the posterior cingulate gyrus to the precuneus is reduced in Alzheimer-type dementia [2]. MRI can also be used to evaluate cerebral blood flow using arterial spin labeling (ASL). ASL reveals reduced blood flow from the posterior cingulate gyrus to the precuneus in patients with Alzheimer-type dementia [3]. In systematic review and meta-analysis, sensitivity, specificity, positive likelihood ratio, negative likelihood ratio, and diagnostic odds ratio of ASL for diagnosing Alzheimer-type dementia was 0.83 (95% CI: 0.78-0.87), 0.81 (95% CI: 0.76-0.86), 4.52 (95% CI: 3.40-6.00), 0.22 (95% CI: 0.17-0.28), and 19.31(95% CI: 12.30-30.31) for Alzheimer-type dementia diagnosis by ASL [4]. Furthermore, hippocampal atrophy is a characteristic index of Alzheimer-type dementia [5]. A comparison method using a database of healthy participants is used to evaluate hippocampal atrophy using MRI. The voxel-based specific regional analysis system for Alzheimer’s disease (VSRAD) includes a database of healthy participants that can be used to quantify the degree of hippocampal atrophy in individual cases. The VSRAD establishes a region of interest (ROI) around the hippocampus and quantifies the degree of brain atrophy based on a statistical index called the Z-score. The diagnostic performance of the VSRAD for Alzheimer-type dementia is 87% [6]. Moreover, the VSRAD is useful in differentiating Alzheimer-type dementia from dementia with Lewy body (DLB) [7]. However, from these reports, there are two methods: atrophy of the brain (MRI) and brain perfusion (ASL and brain perfusion SPECT).

We have clinical question about the relationship between atrophy of brain and brain perfusion.

Our previous study failed to find a clear relationship between the VSRAD scores and ASL [8], and this relationship remains unclear. Therefore, this study aimed to examine the relationship between VSRAD scores and ASL, particularly the relationship between reduced blood flow in the posterior cingulate gyrus and precuneus, which is considered useful in diagnosing Alzheimer-type dementia.

## 2 METHODS

### 2.1 Study Design

This retrospective, case-control study was conducted at the Iga City General Hospital.

### 2.2 Patient Selection

#### 2.2.1 Inclusion Criteria

This study included patients with suspected dementia, according to their institution or nearby medical clinics. Brain magnetic resonance angiography (MRA) was performed at the Iga City General Hospital for detailed examination. The results were analyzed using VSRAD from April 1, 2019, to June 30, 2024. Only initial imaging findings were used in cases with multiple imaging examinations performed during the specified period. This study is an extension of a previous one and includes some data from that study [8].

#### 2.2.2 Exclusion Criteria

The exclusion criteria were as follows: (1) insufficient VSRAD analysis, (2) defective MRA or T2-weighted/fluid-attenuated inversion recovery (FLAIR) image, (3) progressive supranuclear palsy, (4) wallerian degeneration, (5) arachnoid cyst, (6) deformation caused by brain bleeding, (7) acute cerebrovascular disease (cerebral hemorrhage, cerebrovascular infarction, or hematoma), (8) brain tumor, (9) artifact, and (10) incomplete termination.

### 2.3 Magnetic Resonance Imaging

All patients underwent brain MRI using an Ingenia 1.5 T (Philips, Amsterdam, The Netherlands) at Iga City General Hospital, Mie, Japan. MRI included diffusion-weighted (b=0 and b=1000), T1-weighted, T2-weighted, FLAIR, susceptibility-weighted imaging (SWI), ASL, and MRA images.

### 2.4 Arterial Spin-labeling Value

MRI images were evaluated by a radiologist (YO) certified by the Board of Radiation Oncology and Nuclear Medicine with experience in VSRAD research [8] and research teaching [9]. ASL was evaluated as follows: (1) the ROI was set at the posterior cingulate and precuneus (almost the same slice at the lateral ventricle), and (2) the maximum value of ASL was calculated at two points (upper and lower).

The visual evaluation included (1) micro bleeding (SWI images), (2) Deep and Subcortical White Matter Hyperintensity (DSWMH), (3) Periventricular Hyperintensity (PVH), and (4) abnormal MRI. The DSWMH and PVH score is based on the report by Shinohara [10]. and information from online sources regarding radiology imaging [11]. Microbleeding was defined as the presence of a single microhemorrhage. The labeling time for ASL was set to 1,800 ms.

### 2.5 Voxel-based Specific Regional Analysis System for Alzheimer’s Disease

A VSRAD advance (Eisai Co., Ltd., Tokyo, Japan) was used. The accompanying text in the software was used as a reference for the trial. The volume of interest (VOI), or ROI, was the medial temporal region (hippocampus, parahippocampal gyrus, and par hippocampal gyrus).

The VSRAD uses measures of atrophy within the VOI, percentage of atrophic regions within the VOI, percentage of total brain atrophy, atrophy ratio (ratio of the percentage of atrophy in the target ROI to that of the whole brain), gray matter dorsal brainstem VOI atrophy, gray matter dorsal brainstem VOI atrophy, and white matter dorsal brainstem VOI atrophy.

Based on the literature [7], atrophy ≥ 2 in the VOI was classified as Alzheimer-type dementia, and atrophy < 2 in the VOI and ≥ 0.2 in the gray matter dorsal brainstem VOI and atrophy ≥ 0.2 in the white matter dorsal brainstem VOI was classified as DLB. The “other” group included patients who did not meet either of the above criteria.

### 2.6 Statistical Analyses

We evaluated the correlation between the ASL value at the posterior cingulate precuneus and the degree of atrophy within the VOI, percentage of atrophic areas within the VOI, atrophy ratio, percentage of total brain atrophy, and age.

The analysis was performed using EZR developed by the Omiya Medical Center of Jichi Medical University [12]. Mann–Whitney U tests were used to compare the two groups. A receiver operating characteristic (ROC) analysis was used to determine the cutoff values. The correlation between the two variables was evaluated using Spearman’s rank correlation coefficient. A logistic model was used for single-variate analysis and multi-variate analysis. For multivariate analysis, we used stepwise method. P<0.05 was set as the level of statistical significance.

### 2.7 Ethical Considerations

This study was conducted with permission from the Ethics Committee of Iga City General Hospital (Nos. 1044 and 277).

We used the opt-out method and written informed consent was waived. Opt-out information for this study was posted on the hospital website.

## 3 RESULTS

A total of 39 patients were excluded, resulting in 519 patients being included in this study.

### 3.1 Alzheimer-type Dementia Group

A total of 179 patients were observed in this group (55 men and 124 women), aged 82.8±5.7 years. The mean value of degree of atrophy within the VOI was 2.95±0.87, the percentage of atrophic areas within the VOI was 64.8±17.5, the atrophy ratio was 10.1±4.5, the percentage of total brain atrophy was 7.48±3.26, the inter-VOI shrinkage of the dorsal gray matter brainstem was 0.64±0.25, and the inter-VOI atrophy of the dorsal white matter brainstem was 0.32±0.41. Microbleeding was observed in 25 patients. The mean values of ASL at the posterior cingulate and precuneus were 77.0±24.4 mL/min/100 g and 77.3±25.2 mL/min/100 g. The distribution of DSWMH scores was 5, 35, 72, 48, and 15 at scores 0, 1, 2, 3, and 4, respectively. The distribution of PVH scores was 4, 35, 70, 50, and 16 at scores 0, 1, 2, 3, and 4, respectively. These results are shown in Table **1**.

In all patient groups, the correlation coefficient between the atrophy ratio and ASL 1 in the posterior cingulate and precuneus was statistically significant (correlation coefficient=–0.176, P=0.02). The correlation coefficients between the percentage of total brain atrophy and ASL 1/ASL 2 in the posterior cingulate and precuneus were statistically significant (correlation coefficient=–0.212, P<0.01; correlation coefficient=–0.200, P<0.01). The correlation coefficients between the percentage of total brain atrophy and ASL 1/ASL 2 in the posterior cingulate and precuneus were statistically significant (correlation coefficient=–0.212, P=0.02; correlation coefficient=–0.206, P=0.03). The correlation coefficients between the DSWMH and ASL 1/ASL 2 in the posterior cingulate and precuneus were statistically significant (correlation coefficient=–0.245, P<0.01; correlation coefficient=–0.238, P<0.01). The correlation coefficients between the PVH and ASL 1/ASL 2 in the posterior cingulate and precuneus were statistically significant (correlation coefficient=–0.196, P<0.01; correlation coefficient=–0.191, P<0.01). These results are shown in Table **2**.

### 3.2 Dementia with Lewy Body (DLB) Group

A total of 142 patients were observed in this group (65 men and 77 women), aged 79.3±7.79 years. The mean value of the degree of atrophy within the VOI was 1.05±0.45, the percentage of atrophic areas within the VOI was 10.0±12.0, the atrophy ratio was 2.10±3.20, the percentage of total brain atrophy was 5.19±3.22, the inter-VOI shrinkage of the dorsal gray matter brainstem was 0.82±0.29, and the inter-VOI atrophy of the dorsal white matter brainstem was 0.52±0.38. Microbleeding was observed in 37 patients. The mean values of ASL at the posterior cingulate and precuneus were 78.3±81.3 mL/min/100 g and 80.2±23.6 mL/min/100 g, respectively. The distribution of DSWMH scores was 6, 37, 45, 37, and 17 at scores 0, 1, 2, 3, and 4, respectively. The distribution of PVH scores was 6, 37, 45, 39, and 15 at scores 0, 1, 2, 3, and 4, respectively. These results are shown in Table **3**.

In all patient groups, the correlation coefficient between the percentage of total brain atrophy and ASL 1/ASL 2 in the posterior cingulate and precuneus was statistically significant (correlation coefficient=–0.215, P=0.01; correlation coefficient=–0.223). These results are shown in Table **4**.

### 3.3 Other Groups

A total of 198 patients were observed in this group (63 men and 135 women), aged 79.2±7.7 years. The mean value of degree of atrophy within the VOI was 1.20±0.45, the percentage of atrophic areas within the VOI was 14.2±12.9, the atrophy ratio was 3.46±3.84, the percentage of total brain atrophy was 5.00±2.65, the inter-VOI shrinkage of the dorsal gray matter brainstem was 0.79±0.30 dorsal white matter, and the inter-VOI atrophy of the dorsal brainstem was 0.09±0.20. Microbleeding was observed in 35 patients. The mean values of ASL at the posterior cingulate and precuneus were 80.2±22.33 mL/min/100 g and 80.4±22.8 mL/min/100 g, respectively. The distribution of DSWMH scores was 17, 47, 65, 57, and 10 at scores 0, 1, 2, 3, and 4, respectively. The distribution of PVH scores was 16, 47, 65, 57 and 10 at scores 0, 1, 2, 3, and 4, respectively. These results are shown in Table **5**.

In all patient groups, no statistically significant correlation was found between the ASL value in the posterior cingulate precuneus and the degree of atrophy within the VOI, percentage of atrophic areas within the VOI, atrophy ratio, percentage of total brain atrophy, age, DSWMH, and PVH. These results are shown in Table **6**.

### 3.4 Comparison between Alzheimer-type Dementia and Other Groups

No statistically significant difference was observed in ASL 1 and 2 values between Alzheimer-type dementia and other groups (ASL 1: 74.5 mL/min/100 g *vs*. 78.8 mL/min/100 g, P=0.08; ASL 2: 74.8 mL/min/100 g *vs*. 79.2 mL/min/100 g, P=0.101). We compared the Alzheimer-type dementia group and other groups using the non-VSRAD analysis factors, such as age, sex, ASL1, ASL2, bleeding, DSWMH, and PVH by the logistic model. Multivariate analysis shows statically significant value about age [HR(hazard ratio)0.920, 95% CI(confidence interval)0.886-0.955, P<0.01.]. These results are shown in Table **7**.

### 3.5 Comparison between the DLB and other Groups

A statistically significant difference existed in the ASL 1 value between the DLB and other groups (ASL 1: 68.9 mL/min/100 g *vs*. 78.8 mL/min/100 g, P<0.01). However, no statistically significant difference was observed in the ASL 2 value between Alzheimer-type dementia and other groups (ASL 1: 78.4 mL/min/100 g *vs*. 79.2 mL/min/100 g, P=0.47). The DLB group and other groups are compared using the non-VSRAD analysis factors, such as age, sex, ASL1, ASL2, bleeding, DSWMH, and PVH by the logistic model. Multivariate analysis shows statically significant value about sex[HR1.810, 95% CI1.600-.2.820, P<0.0^1^]. These results are shown in Table **8**.

### 3.6 Comparison between the Alzheimer-type Dementia and DLB

No statistically significant difference was observed in ASL 1 and 2 values between the Alzheimer-type dementia and DLB groups (ASL 1: 74.5 mL/min/100 g *vs*. 69.3 mL/min/100 g, P=0.093; ASL 2: 74.8 mL/min/100 g *vs*. 78.9 mL/min/100 g, P=0.258). We compared the Alzheimer-type dementia group and other groups using the non-VSRAD analysis factors, such as age, sex, ASL1, ASL2, bleeding, DSWMH, and PVH by the logistic model. Multivariate analysis shows statically significant value about age [HR0.924, 95%CI 0.891-0.959, P<0.01], sex[HR1.800, 95%CI1.110-2.92, P<0.01], bleeding[HR12.290, 95%CI 1.260-24.140, P<0.01. These results are shown in Table **9**.

## 4 DISCUSSION

This study examined the relationship between the VSRAD and ASL on brain MRI, specifically the relationship between blood flow from the posterior cingulate gyrus and the precuneus. In a previous study comparing 30 patients with Alzheimer-type dementia and 41 controls, the positive diagnostic rate of Alzheimer-type dementia was 87.8% when Z-scores were used [6]. In another study of 30 patients with Alzheimer-type dementia and 40 healthy participants, the VSRAD had a dementia sensitivity of 91.6%. In contrast, the VSRAD had a sensitivity of 86.4% and a specificity of 97.5% for very mild Alzheimer-type dementia [13]. However, the VSRAD can differentiate Alzheimer-type dementia from DLB owing to the different degrees of atrophy in the gray matter and dorsal brainstem, with a sensitivity of 50−56%, a specificity of 68−76%, and a positive predictive value of 63−65% reported for DLB [7]. In contrast, the combination of the VSRAD and e-ZIS (brain perfusion SPECT) improves diagnostic performance compared with MMSE alone in 112 cases of mild dementia and 128 cases of Alzheimer-type dementia [14]. Therefore, although the VSRAD may be relatively inadequate for diagnosing DLB, it demonstrates diagnostic capability for Alzheimer-type dementia.

The VSRAD scores may be useful in assessing the changes associated with the progression of dementia. A study involving 72 patients (15 with Alzheimer-type dementia) reported that the larger the Z-score (a measure of brain atrophy) of the VSRAD, the poorer the position and memory scores [15]. Another study, including 15 cases, reported that the number of teeth and frequency of exercise decreased as brain atrophy progressed [16]. A comparison of 28 patients with diabetes and 28 controls found that VSRAD scores were higher and whole-brain atrophy was more prominent in older patients with type 2 diabetes than in controls [17]. A cohort study comparing exercise plus music therapy with exercise therapy alone and measuring brain atrophy with the VSRAD reported less cognitive decline in the exercise plus music therapy group than in the exercise therapy alone group [18, 19].

ASL may also be useful in diagnosing Alzheimer-type dementia. Several studies have investigated ASL in diagnosing dementia, including a comparison of 20 patients with Alzheimer-type dementia and 23 normal participants with the ROI in the anterior cingulate gyrus, with an area under the curve of ≤0.9 for differentiation between the two groups [20]. When 17 patients with Alzheimer-type dementia were compared with 19 healthy participants, the diagnostic performance of ASL was comparable to that of 18F-fluorodeoxyglucose-PET [21]. When comparing 71 patients with Alzheimer-type dementia, 35 with mild dementia, and 73 healthy participants, patients with Alzheimer-type dementia had decreased blood flow in the parietal lobes [22]. The cerebral blood flow in Alzheimer’s and Parkinson’s diseases is similar. However, reduced blood flow in the medial parietal lobe is a characteristic of Parkinson’s disease with dementia, whereas reduced blood flow in the right frontal lobe is a characteristic of Alzheimer’s disease [23]. In a study of 148 patients, reduced cerebral blood flow in the posterior cingulate gyrus was helpful in predicting the risk of a shift toward dementia [24].

However, our study showed that the ASL decrease in the posterior cingulate gyrus and precuneus was not necessarily a characteristic phenomenon in the Alzheimer-type and DLB groups and that the ASL decrease in the posterior cingulate gyrus and precuneus also occurred in the other groups. Furthermore, ASL values in the posterior cingulate gyrus and precuneus depend on certain factors. In our study, the correlation coefficient of the ASL values in the posterior cingulate gyrus and precuneus with the percentage of atrophic areas within the VOI was statistically significant in both the Alzheimer-type dementia group and the DLB-type dementia group. Conversely, in the other group, no statistically significant correlation was found between the ASL value in the posterior cingulate precuneus and the degree of atrophy within the VOI, percentage of atrophic areas within the VOI, atrophy ratio, percentage of total brain atrophy, age, DSWMH, and PVH.

Additionally, the correlation values between ASL in the posterior cingulate gyrus and precuneus and the parameters were small (r=0.2–0.3). We believe that the blood flow in the posterior cingulate gyrus and precuneus is not necessarily a specific indicator of dementia. In DLB, the cingulate island sign refers to the relative preservation of blood flow in the posterior cingulate gyrus compared to the surrounding occipital lobe and is considered a characteristic imaging feature of the disease. Cerebral blood flow SPECT can be used to diagnose Alzheimer-type dementia and DLB, with a sensitivity of 84.6%, a specificity of 84.6%, and a positive predictive value of 84.6% [25]. Cutoff values for cerebral blood flow SPECT using software have also been reported [26]. However, reports indicate that the specificity is low [27], and the cingulate island sign is less likely to appear in older adults [28]. Although comparing the results of this study, conducted using MRI, with those of brain perfusion SPECT is challenging, we found that decreased blood flow in the posterior cingulate gyrus and precuneus tended to appear with an increasing percentage of total brain atrophy areas in the DLB group on the VSRAD and in the other group. Based on these results, decreased blood flow in the posterior cingulate gyrus and precuneus appears to be one of the weakest indicators of dementia. We think that it is difficult to classify the probability of dementia or dementia diagnosis based on only the degree of cerebral blood flow reduction.

This study has some limitations. First, this was a retrospective study, and the psychological scores and other assessments were insufficient. Second, this study involved a visual evaluation; therefore, subjectivity could not be eliminated. Third, the comorbid conditions, medication, psychological scores and other assessments were insufficient.

Fourth, there is a lack of control group. Also, we evaluated only 1.5T MRI and not 3.0T MRI. Finally, clinical information was insufficient; therefore, prospective studies with larger sample sizes are warranted.

In the future, we plan to include more clinical information as a secondary analysis, comparing the results with clinical information and, if possible, comparing the results with the quantitative values of ASL Moreover, a comparison of VSRAD/ASL and amyloid/tau PET (positron emission CT) is necessary.

## CONCLUSION

Blood flow reduction in the posterior cingulate gyrus and precuneus is not necessarily a characteristic phenomenon in the Alzheimer’s and DLB groups and is influenced by several factors. Therefore, it is necessary to avoid using cerebral blood flow assessment alone when diagnosing dementia.

## Figures and Tables

**Table 1:** Patient characteristics in the Alzheimer-type dementia group.

**Factor**	**Value**
Number of cases	179
Age (years)	82.8±5.7
Sex, number	-
Male	55
Female	125
Degree of atrophy within the VOI	2.95±0.87
Percentage of atrophic areas within the VOI	64.8±17.5
Atrophy ratio	10.1±4.5
Percentage of total brain atrophy	7.48±3.26
Inter-VOI shrinkage of the dorsal gray matter of the brainstem	0.64±0.25
Inter-VOI shrinkage of the dorsal white matter of the brainstem	0.32±0.41
SWI, number	-
No bleeding	148
Bleeding	25
NA	5
ASL at the posterior cingulate and precuneus (mL/min/100 g)	-
ASL 1	77.0±24.4
ASL 2	77.3±25.2
DSWMH	Score 0:5 Score1:35 Score2:72 Score3:48 Score5:15
PVH	Score 0:4 Score1:35 Score2:70 Score3:50 Score5:16

**Notes:** Data are presented as means±standard deviations unless otherwise described. **Abbreviations:** VOI, volume of interest; SWI, susceptibility-weighted; ASL, arterial spin labeling.

**Table 2:** Correlations between ASL and VSRAD/age factors in the Alzheimer-type dementia group.

	**ASL 1 at the Posterior Cingulate and Precuneus**		**ASL 2 at the Posterior Cingulate and Precuneus**	
	**r coefficient**	**P-value**	**r coefficient**	**P-value**
Degree of atrophy within the VOI	–0.107	0.164	–0.149	0.053
Percentage of atrophic areas within the VOI	–0.042	0.587	–0.082	0.289
Atrophy ratio	0.176	0.02	0.148	0.054
Percentage of total brain atrophy	–0.214	<0.01	–0.189	<0.01
Age	–0.09	0.284	–0.116	0.131
DSWMH	-0.245	<0.01	-0.238	<0.01
PVH	-0.196	<0.01	-0.191	<0.01

**Notes:** P<0.05 was considered statistically significant. **Abbreviations:** ASL: arterial spin labeling; VSRAD: voxel-based specific regional analysis system for Alzheimer’s disease; VOI: volume of interest; DSWMH, Deep and Subcortical White Matter Hyperintensity; PVH: Periventricular Hyperintensity.

**Table 3:** Patient characteristics in the dementia with Lewy body group.

**Factor**	**Value**
Number of cases	142
Age (years)	79.3±7.79
Sex, number	-
Male	65
Female	125
Degree of atrophy within the VOI	1.05±0.45
Percentage of atrophic areas within the VOI	10.0±12.0,
Atrophy ratio	2.10±3.20
Percentage of total brain atrophy	5.19±3.22
Inter-VOI shrinkage of the dorsal gray matter of the brainstem	0.82±0.29
Inter-VOI shrinkage of the dorsal white matter of the brainstem	0.52±0.38
SWI, number	-
No bleeding	105
Bleeding	37
ASL at the posterior cingulate and precuneus (mL/min/100 g)	-
ASL 1	78.3±81.3
ASL 2	80.2±23.6
DSWMH	Score 0:6 Score1:37 Score2:45 Score3:37 Score5:17
PVH	Score 0:6 Score1:37 Score2:45 Score3:39 Score5:15

**Note:**Data are presented as means±standard deviations unless otherwise described. **Abbreviations:** VOI: volume of interest; SWI: susceptibility-weighted; ASL: arterial spin labeling; DSWMH: Deep and Subcortical White Matter Hyperintensity; PVH: Periventricular Hyperintensity.

**Table 4:** Correlations between ASL and VSRAD/age factors in the dementia with Lewy body group.

	**ASL 1 at the posterior cingulate and precuneus**		**ASL 2 at the posterior cingulate and precuneus**	
	**r coefficient**	**P-value**	**r coefficient**	**P-value**
Degree of atrophy within the VOI	–0.02	0.804	–0.038	0.653
Percentage of atrophic areas within the VOI	–0.007	0.933	-0.059	0.488
Atrophy ratio	0.058	0.53	0.010	0.900
Percentage of total brain atrophy	–0.215	<0.01	–0.233	<0.01
Age	–0.157	0.062	–0.063	0.459
DSWMH	-0.05	0.528	-0.122	0.151
PVH	-0.064	0.454	-0.128	0.13

**Note:** P<0.05 was considered statistically significant. **Abbreviations:** ASL: arterial spin labeling; VSRAD: voxel-based specific regional analysis system for Alzheimer’s disease; VOI: volume of interest; DSWMH: Deep and Subcortical White Matter Hyperintensity; PVH: Periventricular Hyperintensity.

**Table 5:** Patient characteristics in the “other” group.

**Factor**	**Value**
Number of cases	198
Age (years)	79.2±7.7
Sex, number	-
Male	63
Female	135
Degree of atrophy within the VOI	1.20±0.45
Percentage of atrophic areas within the VOI	14.2±12.9
Atrophy ratio	3.46±3.84
Percentage of total brain atrophy	5.00±2.65
Inter-VOI shrinkage of the dorsal gray matter of the brainstem	0.79±0.30
Inter-VOI shrinkage of the dorsal white matter dorsal of the brainstem	0.09±0.20
SWI, number	-
No bleeding	159
Bleeding	35
Other	4
ASL at the posterior cingulate and precuneus (mL/min/100 g)	-
ASL 1	80.2±22.3
ASL 2	80.4±22.8
DSWMH	Score 0:17 Score1:47 Score2:65 Score3:57 Score4:10
PVH	Score 0:16 Score1:47 Score2:65 Score3:57 Score5:10

**Note:** Data are presented as means±standard deviations unless otherwise described. **Abbreviations:** VOI: volume of interest; SWI: susceptibility-weighted; ASL: arterial spin labeling; DSWMH: Deep and Subcortical White Matter Hyperintensity; PVH: Periventricular Hyperintensity.

**Table 6:** Correlation value between ASL and VSRAD/age factors in the “other” group.

	**ASL 1 at the Posterior Cingulate and Precuneus**		**ASL 2 at the Posterior Cingulate and Precuneus**	
	**R coefficient**	**P-value**	**R coefficient**	**P-value**
Degree of atrophy within the VOI	–0.046	0.528	–0.060	0.441
Percentage of atrophic areas within the VOI	–0.046	0.524	–0.062	0.376
Atrophy ratio	–0.057	0.3433	–0.060	0.402
Percentage of total brain atrophy	–0.028	0.697	–0.069	0.343
Age	-0.083	0.255	–0.128	0.076
DSWMH	-0.084	0.252	–0.013	0.860
PVH	-0.096	0.200	–0.022	0.766

**Note**: P<0.05 was considered statistically significant. **Abbreviations:** ASL: arterial spin labeling; VSRAD: voxel-based specific regional analysis system for Alzheimer’s disease; VOI: volume of interest; DSWMH: Deep and Subcortical White Matter Hyperintensity; PVH: Periventricular Hyperintensity.

**Table 7:** Comparison Alzheimer-type dementia and other groups factor.

	**HR**	**95%CI**	**P value**	**HR**	**95%CI**	**P value**
Age	0.919	0.887-0.951	<0.01	0.920	0.886-0.955	<0.01
Sex	1.050	0.680-1.630	0.819	-	-	-
ASL1	1.010	0.997-1.010	0.199	-	-	-
ASL2	1.010	0.997-1.010	0.199	-	-	-
Bleeding	1.310	0.750-2.300	0.342	-	-	-
DSWMH	0.811	0.659-0.996	0.046	5.930	0.643-1.490	0.12
PVH	0.789	0.641-0.972	0.026	0.164	0.018-1.490	-

**Table 8:** Comparison of DLB and other groups.

**Factor**	**HR**	**95%CI**	**P value**	**HR**	**95%CI**	**P value**
age	1.000	0.975-1.030	0.868	-	-	-
sex	1.810	1.160-2.820	<0.01	1.810	1.160-2.820	<0.01
ASL1	0.999	0.995-1.000	0.759	-	-	-
ASL2	1.000	0.990-1.010	0.950	-	-	-
bleeding	1.600	0.948-2.700	0.078	-	-	-
DSWMH	1.170	0.953-1.440	0.133	-	-	-
PVH	1.1500	0.933-1.420	0.190	-	-	-

**Table 9:** Comparison of Alzheimer-type dementia and other groups.

Factor	HR	95%CI	P value	HR	95%CI	P value
age	0.93	0.89- 0.959	<0.01	0.924	0.891-0.959	<0.01
sex	1.90	1.200-3.010	<0.01	1.800	1.110-2.920	<0.01
ASL1	1.00	0.997-1.000	0.848	-	-	-
ASL2	1.00	0.996-1.010	0.30	-	-	-
bleeding	2.100	1.190-3.700	0.01	2.290	1.260-4.140	<0.01
DSWMH	0.967	0.776-1.210	0.767	-	-	-
PVH	0.920	0.737-1.150	0.466	-	-	-

## Data Availability

All data generated or analyzed during this study are included in this published article.

## LIST OF ABBREVIATIONS

ASL: Arterial Spin Labeling
VOI: Volume of Interest
DLB: Dementia with Lewy Bodies
MMSE: Mini-Mental State Examination
CT: Computed Tomography
MRI: Magnetic Resonance Imaging
SPECT: Single-photon Emission Computed Tomography
PET: Positron Emission Tomography
VSRAD: Voxel-based Specific Regional Analysis System for Alzheimer’s Disease
MRA: Magnetic Resonance Angiography
SWI: Susceptibility-weighted Imaging
DSWMH: Deep and Subcortical White Matter Hyperintensity
PVH: Periventricular Hyperintensity

## STANDARDS OF REPORTING

STROBE guidelines were followed.
